# Thermally Triggered Mechanically Destructive Electronics Based On Electrospun Poly(ε-caprolactone) Nanofibrous Polymer Films

**DOI:** 10.1038/s41598-017-01026-6

**Published:** 2017-04-19

**Authors:** Yang Gao, Kyoseung Sim, Xin Yan, Jiang Jiang, Jingwei Xie, Cunjiang Yu

**Affiliations:** 1 Department of Mechanical Engineering, University of Houston, Houston, TX 77204 USA; 2 School of Mechanical and Power Engineering, East China University of Science and Technology, Shanghai, 200237 China; 3 Materials Science and Engineering Program, University of Houston, Houston, TX 77204 USA; 4 Department of Surgery, Mary & Dick Holland Regenerative Medicine Program, University of Nebraska Medical Center, Omaha, NE 68198 USA; 5 Department of Electrical and Computer Engineering, University of Houston, Houston, TX 77204 USA

## Abstract

Electronics, which functions for a designed time period and then degrades or destructs, holds promise in medical implants, reconfigurable electronic devices and/or temporary functional systems. Here we report a thermally triggered mechanically destructive device, which is constructed with an ultra-thin electronic components supported by an electrospun poly(ε-caprolactone) nanofibrous polymer substrate. Upon heated over the melting temperature of the polymer, the pores of the nanofibers collapse due to the nanofibers’ microscopic polymer chain relaxing and packing. As a result, the polymer substrate exhibits approximately 97.5% area reduction. Ultra-thin electronic components can therefore be destructed concurrently. Furthermore, by integrating a thin resistive heater as the thermal trigger of Joule heating, the device is able to on-demand destruct. The experiment and analytical results illustrate the essential aspects and theoretical understanding for the thermally triggered mechanical destructive devices. The strategy suggests a viable route for designing destructive electronics.

## Introduction

Typically, to achieve long-lasting function is a general goal for the development of electronics, which requires stable operation over time. In some applications including but not limit to reconfigurable circuits, temporary and dissoluble biomedical implants, disposable and degradable electronics, and data-secure hardware systems^1–4^, however, electronic devices are desired to function for a designed certain time period and then to dis-function, degrade or even disappear. Reconfigurable circuits can perform one function for a certain period of time and then others through electrical structure reconfigurations. Temporary and dissoluble medical implants can fulfill their medical diagnostic and therapeutic functions for a certain period of time and then disintegrate or be dissolved in biofluids without surgical removal procedure^5^. Recently, an emerging class of electronics, namely transient electronics, which physically disappears after their usage for certain time frame^4, 6–17^, is developed for those purposes. A wide range of transient electronic devices has been reported, including resistors^2^, transistors^2, 12^, diodes^2^, photodetectors^2^, batteries^16^, energy harvestors^18^, etc. It is noted that the transience of those reported transient electronics mainly relies on chemical dissolution. The materials associated with the aforementioned transient devices comprise of polymers, such as silk fibroin, poly(ε-caprolactone) (PCL), and poly(lactic acid) for substrates^12^; metals, such as Mg, Fe, Zn, Al, W, and Mo for electrodes or interconnects^2, 19^; insulators, such as MgO, SiO_2_, and SiN for dielectrics or encapsulation layers^2, 14^; and semiconductors, such as Si^4^, GaAs^18^, and ZnO^8^ as active materials in transistors and optoelectronics.

Due to the chemical dissolution requirement, the material option is very narrow; therefore the device configurations and capabilities are limited. In addition, the operational lifetime of those transient devices strongly depends on their dissolution processes, therefore it is lack of on-demand controllability^2, 6^. While chemical dissolving has been explored, other destruction modes, such as mechanical destruction, has not been explored or reported.

Here we report a novel design of destructive device, which is dissolving solution free. The destruction takes place through mechanical distortion or fragmentation in a controllable manner. As described in the following, devices can be destructed through the shrinkage of the polymer substrate upon thermal triggering. Specifically, the device is constructed with an ultra-thin functional components and electronics supported by an electrospun poly(ε-caprolactone) (PCL) nanofibrous polymer substrate. Upon heated, such electrospun PCL nanofibrous polymer film shrinks immediately. Thin electronic devices bonded on the PCL nanofibrous polymer film become completely deconstructed once deforming concurrently with the substrate shrinkage. In addition, on-demand self-destruction is achieved by integrating a thin resistive heater as the source of Joule heating trigger. We outline the materials preparation, device construction, and provide examples of controllable destruction of thin electronic devices to illustrate all essential aspects of the self-destructive devices.

## Result and Discussion

The electrospun PCL nanofibrous polymer films were used as the substrates for the thermally triggered mechanically destructive electronics. Supplementary Fig. S1 shows the experimental setup for the synthesis of the electrospun PCL nanofibrous polymer films. While a high electrical field is applied to a polymer solution through a metallic needle, the polymer solution is stretched into a fine jet. After the solvent evaporates, nanofibers can be formed and deposited on a collector to form a porous fiber sheet. Such electrospinning allows precise control of fiber dimensions. The properties of the sheet (e.g., mechanical properties and microstructures) can be tailored by varying electrospin parameters including polymer type, collectors, applied electrical field, solution viscosity, injection flow rate, and humidity^20, 21^. More importantly, the electrospun PCL nanofibrous polymer film could undergo large areal shrinkage (~97.5%) in a few seconds upon heated over its melting temperature of ~60 °C^22^, due to the nanofibers’ microscopic polymer chain relaxing and packing and thus macroscopic fiber collapsing. As a result, bonded thin electronic devices on the PCL nanofibrous polymer film become completely deconstructed.

**Figure 1:**
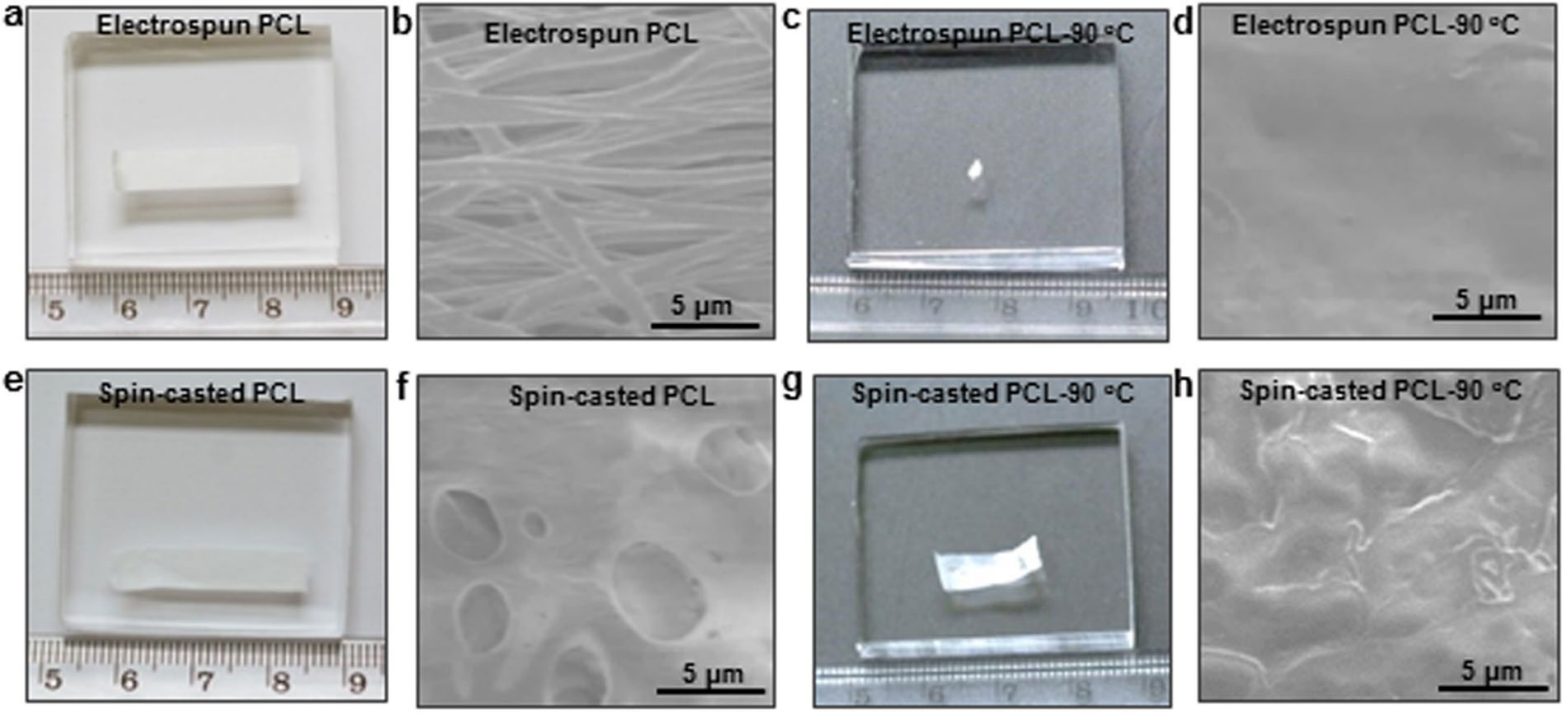
Heat induced shrinkage of electrospun PCL nanofibrous polymer film. (**a**) An optical and (**b**) SEM images of an electrospun PCL nanofibrous polymer film. (**c**) Electrospun PCL nanofibrous polymer film after heated at 90 °C. (**d**) SEM images of the electrospun PCL nanofibrous polymer film after heating. (**e**) Optical and (**f**) SEM images of spin-casted PCL film. (**g**) Spin-casted PCL film after heated at 90 °C. (**h**) SEM images of the spin-casted PCL film after heating.

Figure 1a shows an optical image of an electrospun PCL nanofibrous polymer film (2.5 cm × 0.5 cm) which was produced utilizing a standard electrospinning setup as described elsewhere^23–26^. The thickness of the film is ~120 μm (Supplementary Fig. S2). The scanning electron microscopy (SEM) image of the film made of randomly oriented PCL nanofibers (diameter ~ 500 nm) is shown in Fig. 1b. When heated at the temperature of 90 °C on a hot plate, the electrospun PCL nanofibrous film shrank within 2 sec and turned into a small pellet of less than 2 mm in diameter, as shown in Fig. 1c. Supplementary Fig. S3 shows the squential images to illustrate the time-lapse shrinkage process. The area reduction (before: 1.25 cm^2^; after: 0.0315 cm^2^) of the electrospun PCL film is about ~97.5%. The transformation from nanofibrous meshwork to bulk solids is clearly indicated in Fig. 1b,d. In comparison, a spin-casted PCL film, as shown in Fig. 1e, with the same size as that of the electrospun PCL nanofibrous polymer film was also tested. Pore structure is observed, as exhibited in Fig. 1f. The spin-casted PCL film shrank slowly over a longer period of time (~13 sec), and only about 50% area reduction was observed (Fig. 1g). The nonporous structure of the spin-casted PCL film after heating is shown in Fig. 1h.

To further investigate the fast and large shrinkage phenomenon of the electrospun PCL nanofibrous polymer, SEM images (Supplementary Fig. S4) of the electrospun PCL nanofibrous polymer film heated for different period of time were taken to observe the shrinkage process. As shown in Supplementary Fig. S4b, after the polymer was exposure to heat for 0.5 sec, the straight PCL nanofibers underwent shrinkage and relaxation. After continuous heating for 2 sec, the PCL nanofibers are melted together to form a bulk solid. Electrospun nanofibers are typically composed of stretched non-crystalline molecular chains^27^, which undergo chain relaxation and packing and thus macroscopic shrinkage upon thermal stimulation^28–30^. The microscopic chain relaxation and packing attribute to the fast and dramatic shrinkage.

**Figure 2:**
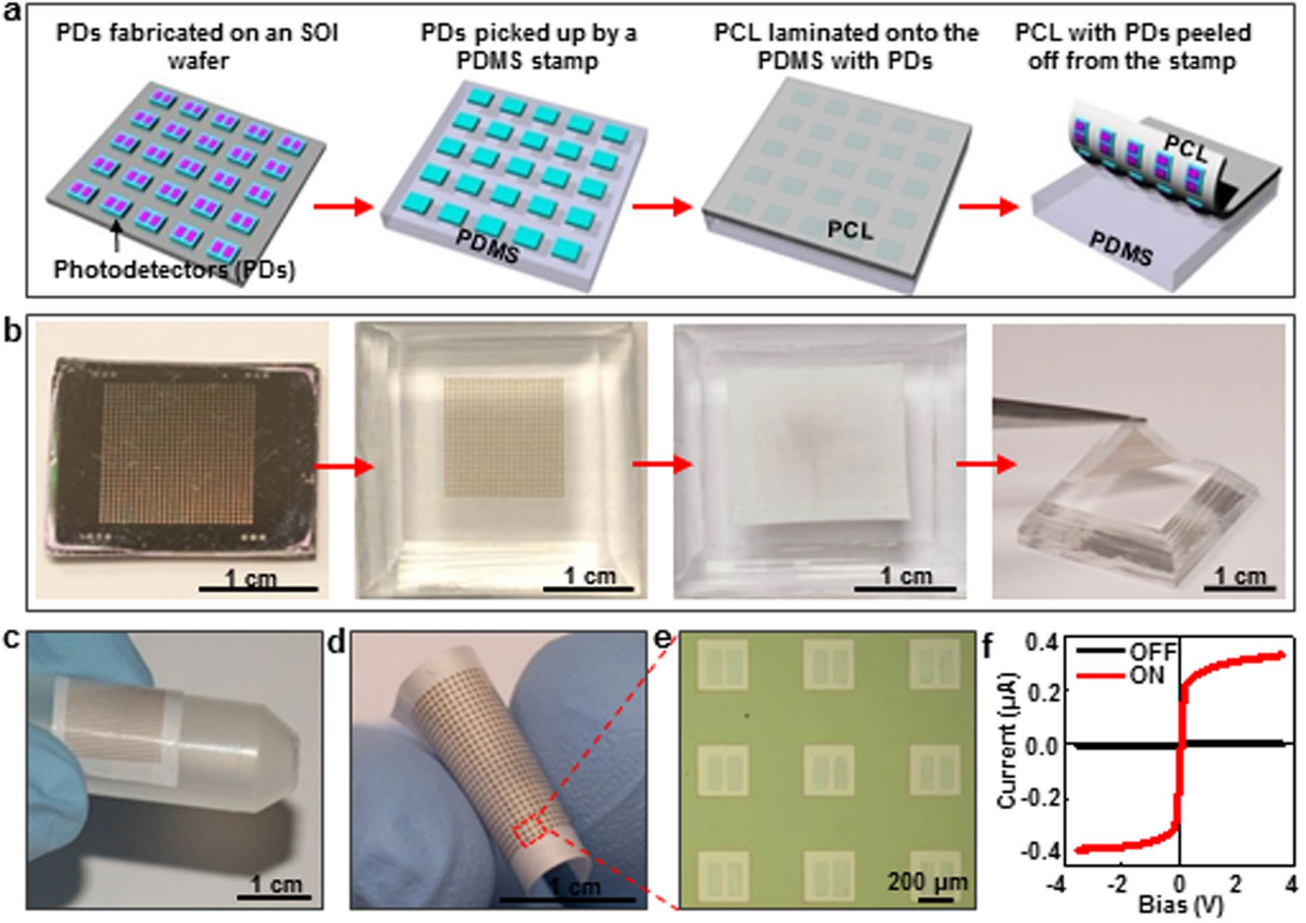
Fabircation and characterization of Si-based photodetectors on electrospun PCL nanofibrous polymer film. (**a**) Schematics and (**b**) photographs illustrating the fabrication process of Si-based photodetectors on an electrospun PCL nanofibrous polymer film. (**c**,**d**) Photographs demonstrating the flexibility of the device. (**e**) Optical image of the photodetector arrays. (**f**) Device performance of the photodetectors on the electrospun PCL nanofibrous polymer film.

Ultra-thin electronics on an electrospun PCL nanofibrous polymer substrate can be mechanically destructed upon heating above the substrate’s melting temperature. As a demonstration, ultra-thin Si (1.25 μm thick) photodetector array was prepared and bonded with the electrospun PCL nanofibrous polymer film. The schematic fabrication and integration procedures are illustrated in Fig. 2a. Specifically, Si-based photodetector array was fabricated from a silicon-on-insulator (SOI) wafer by standard photolithography, reactive ion etching, and adding temporary tethers on the sides to hold their position during undercut etching of SiO_2_. The photodetector array was transfer printed onto an electrospun PCL nanofibrous polymer film using a polydimethylsiloxane (PDMS) stamp. Similar transfer printing technique has been extensively used for constructing inorganic flexible and stretchable electronics^31–33^. To facilitate the bonding between the photodetector array and substrate, a very thin layer of PCL solution served as adhesive was spin-casted on the inked PDMS stamp with photodetectors on. The stamp with adhesive on was then baked at 80 °C for 1 min in an oven to soften the spin-casted adhesive and followed by laminating against and bonding to a piece of electrospun PCL nanofibrous polymer film to complete the integration. Finally, the whole device was peeled off from the stamp. Figure 2b shows sequential optical images corresponding to the schematic process in Fig. 2a, where a 27 × 27 array of 1.25 µm thick Si photodetectors originally fabricated on the SOI wafer was completely transferred onto the electrospun PCL nanofibrous polymer film, at a yield of 100%. Since the PCL film is very thin, the integrated device is very flexible. As shown in Fig. 2c,d, no interfacial delamination or Si photodetector fracture while bent happened. Figure 2e shows an optical image of the photodetector array transferred onto the electrospun PCL substrate. The size of the photodetector is 300 μm × 300 μm. The photo response of the Si-based photodetectors on the electrospun PCL substrate is shown in Fig. 2f. At 3.5 V bias, the photocurrents under illumination and in dark are 0.329 μA and 0.293 nA, respectively. The calculated current ratio under illumination and dark is ~1.1 × 10^3^ at 3.5 V bias, based on the equation *R* = *I*_*bright*_ */I*_*dark*_.

**Figure 3:**
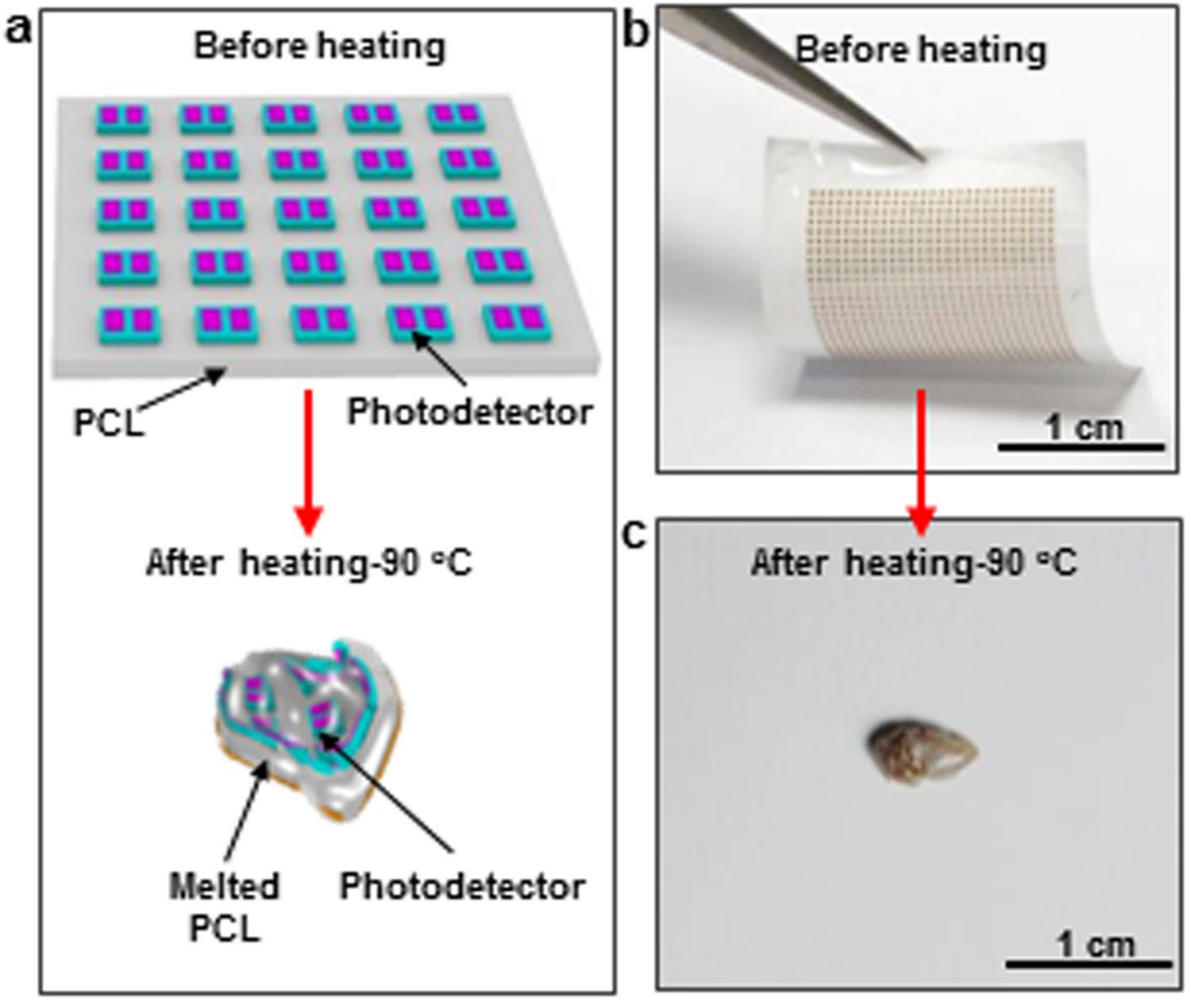
Themral triggered destruction of Si-based photodetectors on electrospun PCL nanofibrous polymer film. (**a**) Schematic illustration of the destruction behavior of Si-based photodetectors on an electrospun PCL nanofibrous polymer film triggered by heating. Photographs of the Si-based photodetectors on an electrospun PCL nanofibrous polymer film (**b**) before and (**c**) after heating.

The destruction of the device upon heating was carefully investigated. The integrated device was heat-treated on a hot plate at the temperature of 90 °C to investigate the destruction behavior of the device as schematically shown in Fig. 3a. The overall size of the integrated photodetector array on an electrospun PCL nanofibrous polymer film is about 1.7 cm × 1.7 cm (Fig. 3b). Upon heated, the PCL nanofibrous membrane shrank dramatically and melted with the photodetectors embedded inside or attached on its surface in a completely distorted manner. Figure 3c shows the optical image of the destructed device. If interconnected, the photodetector array as an imaging device, obviously lose it imaging capability.

**Figure 4:**
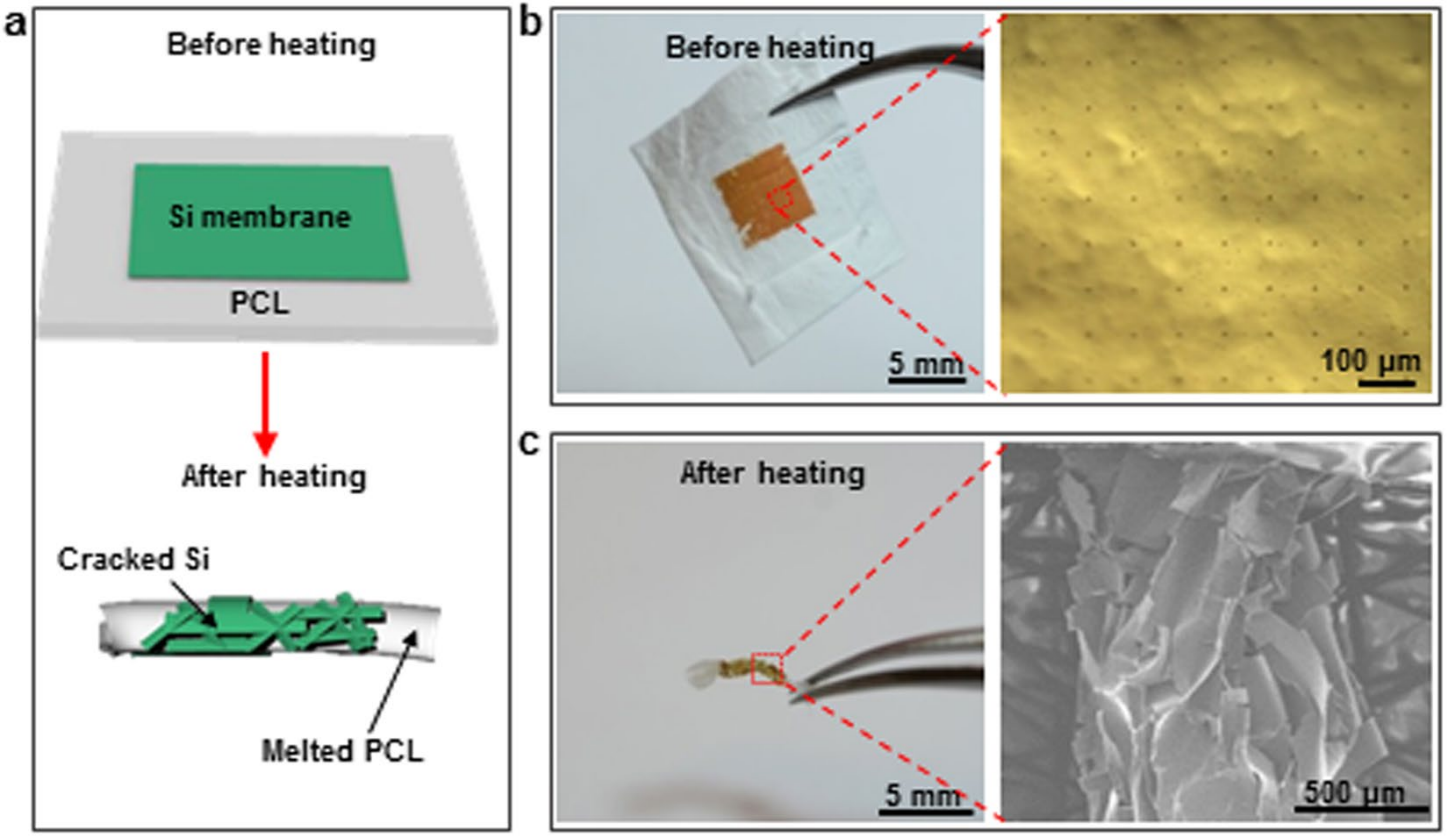
Themral triggered destruction of Si membrane on electrospun PCL nanofibrous polymer film. (**a**) Schematic illustration of the destruction behavior of Si membrane on an electrospun PCL nanofibrous polymer film triggered by heating. Images of the Si membrane on an electrospun PCL nanofibrous polymer film (**b**) before and (**c**) after heating.

Fast and dramatic shrinkage of the electrospun PCL nanofibrous polymer films not only easily induces the device distortion, but also enables fragmentation of thin film devices. Fragmentation, the process of being broken into small and separate parts, typically takes place when thin films, especially brittle films, are stretched to release deformation energy^34^. Such process is irreversible and usually considered to be detrimental to materials and structure failure. Fragmentation of thin electronics, which is made from brittle semiconductor materials, such as Si, could represent a mechanically destructive mode for destructive devices. However, in this study, instead of stretching, the fragmentation of thin brittle films takes place upon dramatic and fast shrinkage of the supporting substrate of electrospun PCL nanofibrous film, as schematically illustrated in Fig. 4a. A 300 nm thick Si membrane (5 mm × 5 mm) was transfer printed on an electrospun PCL nanofibrous polymer film (15 mm × 17 mm). The detailed fabrication processes of the Si membrane (Supplementary Fig. S5) can be found in *Experimental Section*. The destruction of the Si membrane on the electrospun PCL nanofibrous polymer film (Fig. 4b) was studied. Upon heated at 90 °C on a hot plate, the electrospun PCL film shrank immediately, and the Si membrane was fragmentized into tiny (around 1 μm) pieces as shown in Fig. 4c. Thinner membranes, such as 100 nm thick Si, were also successfully fragmentized, as shown in Supplementary Fig. S6.

Such mechanical destructive behavior can also be understood based on the quantitatively analysis of associated mechanics. The fact of shrinking the PCL nanofibrous film with the Si membrane on closely mimics the case of releasing the pre-strain of an elastomer with a Si membrane bonded on the surface^35^. In particular, releasing the pre-strain of an elastomer will induce wrinkling in Si membrane. Once the strain in the Si film associated with the wrinkling (tensile and compressive strain near the peak and valey, corresspondingly) exceeds the fracture limit, fracture happens. We hypothesize that this scenario is responsible for the self-destruction mechanism of the PCL nanofibrous film and Si membrane system. Based on the analytical studies from Jiang *et al*., the maximium strain in the Si film is

1 $${\varepsilon }_{max}=2\sqrt{{\varepsilon }_{pre}{\varepsilon }_{c}}\frac{{(1+\xi )}^{1/3}}{\sqrt{1+{\varepsilon }_{pre}}}$$

where *ε*_*pre*_ is the pre-strain on the substrate, and ξ = 5 *ε*_*pre*_ (1 + *ε*_*pre*_)/32, and ε_c_ is the critical strain on releasing substrate that wrinkling will start to appear, can be described as following

2 $${\varepsilon }_{c}=\frac{1}{4}\,{(\frac{3{E}_{sub}}{{E}_{Si}})}^{2/3}$$

*E*_*sub*_ and *E*_*Si*_ are the modulus of the substrate and Si membrane respectively. Due to the several orders difference in their modulus of the polymer substrate and the Si membrane, ε_c_ is typically smaller than 0.1%. In other words, the Si membrane wrinkles immediately as soon as the pre-strain started to be released. Upon further releasing, the wrinkling wavelength and amplitude change to accomodate until the maximum strain in the Si reaches the fracture limit. To ensure that Si membrane would not fracture (*ε*_*max*_ < 1%)^35^, based on Eq. (1), the calculated allowable maximum *ε*_*pre*_ is 5.3%, where the *E*_*sub*_ and *E*_*Si*_ are 3.8 MPa and 130 GPa, respectively. The modulus of the nanofibrous film was obrained from tensile testing based on a tensile tester (ESM301, Mark-10 Corp), as shown in Supplementary Fig. S7. Since the PCL nanofibrous film experience much larger linear shrinkage than the allowable pre-strain, the Si membrane will fracture upon thermal trigger. While relatively thin Si membranes (<2 μm) have shown similar fragementation behavior. It is noted that, using the polymer film with uniform thickness (~120 μm), much thicker Si membrane, such as a few to tens microns does not show such fragmentation behavior, since the driving force from the PCL shrinkage is not high enough to actuate the Si membrane. Employing thick PCL nanofibrous films suggests a route to destruct relatively thicker Si membranes. Overall, these analysis suggests the thermally triggered mechanical destruction as a feasible approach for destructive devices.

**Figure 5:**
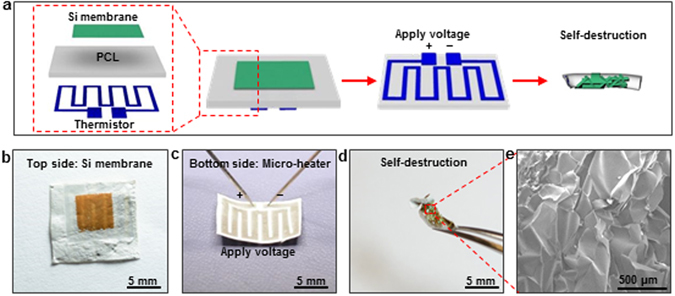
On-demand destruction of Si membrane on electrospun PCL nanofibrous polymer film. (**a**) Schematic illustration of the on-demand self-destruction of Si membrane on an electrospun PCL nanofibrous polymer film. (**b**,**c**) Optical images of both sides of a self-destructive device consisting of an integrated resistor with the electrospun PCL nanofibrous polymer film and Si membrane. (**d**) Optical image of a self-destructed device after applying electrical power on the resistor. (**e**) An SEM image of the fragmentized Si membrane.

Precise control of the functional time period and then destruction immediately is an important feature of those destructive devices. H. L. Hernandez reported the dissolution of the substrate and thus destruction of the electronics through UV illumination^9^, and C. H. Lee reported wirelessly controlled releasing of etching solution in microfluidic system for on-demand chemical dissolution^15^. Thermal induced shrinkage of the electrospun PCL based electronics can also be implemented with on-demand destruction ability. While these above mentioned devices that were destructed with the facilitation of external heating sources, i.e. hotplates, an on-demand self-destructive device exploits structure that involves an integrated resistor, the electrospun PCL nanofibrous polymer film, and electronic devices. As a proof-of-concept, a thin metal resistor and a Si membrane lays on each sides of the electrospun PCL nanofibrous polymer film, as illustrated in Fig. 5a. The integrated device is designed to be destructed on-demand upon passing electrical current through the resistor to induce joule heat and thus trigger fast shrinking and melting of the PCL film. Figure 5a schematically depicts the device configuration and on-demand destruction process. The resistor was fabricated by electron beam deposition of a layer of 1.4 μm thick silver (Ag) on the PCL film through a custom-made shadow mask. The details of the shadow mask fabrication were presented in *Experimental Section*. The resistance of the resistor is ~70 Ω. The resistor is designed in zigzag configuration in order to provide uniform heat on the whole PCL film. The success of PCL film shrinkage resulting from the resistive heating is evidenced by the squential images in Supplementary Fig. S8. A 300 nm thick Si membrane (5 mm × 5 mm) was then transfer printed on the other side of the PCL film. Figure 5b,c show the optical images of both sides of the devices, i.e. the transfer printed Si membrane and resistor, respectively. On-demand destruction of the device was triggered by the application of 100 V on the resistor. The shrinking and melting process completed within 2 sec. During the shrinking process, the integrated heater concurrently deforms with electrospun PCL nanofibrous film. Since the heat can be generated rapidly, even the heater circuit is disconnected, the remaining heat is able to allow the shrinkage process to continue. As shown in Fig. 5d, due to the fast shrinkage triggered by heating, the Si membrane on the PCL film was completely fragmentized (Fig. 5e). These results suggest an alternative on-demand self-destructive mode for destructive electronics.

## Conclusions

In summary, thermally induced fast and dramatic shrinkage of electrospun PCL nanofibrous polymer films enables mechanical destruction of electronics. Experiment illustrates the key operational characteristics of the integrated destructive thin Si devices. The strategy appears appropriate and useful for broad range of destructive electronic devices, including communication circuits, memory, power sources, etc. Although the destructive devices described here are based on electrospun PCL nanofibrous polymer films as substrates, other types of polymer materials with appropriate shrinkage characteristics could extend the variety of device configurations. The results suggest that such thermally triggered mechanical destruction concept and on-demand self-destruction strategy could be useful in emerging reconfigurable electronics and temporary functional components, where mechanical destruction could serve as an effective destructive mode with precise control of the turning point from function to destruction.

## Methods

### Electrospun nanofibrous PCL polymer film preparation

PCL particles (Mw = 80 kDa, Sigma-Aldrich, St. Louis, MO, USA) were dissolved in a solvent mixture consisting of dichloromethane (DCM, Fisher Chemical, Waltham, MA, USA) and N,N-dimethylformamide (DMF, Fisher Chemical, Waltham, MA, USA) at the ratio of 4:1 (v/v) at a concentration of 10% (w/v). The obtained PCL solution was pumped at a flow rate of 0.6 mL h^−1^ using a syringe pump while an electrical potential of 12 kV was applied between a spinneret (a 22-gage needle) and a grounded stainless steel drum collector located 12 cm apart from the spinneret. Nanofibers were collected by the drum collector which rotated at the rate of about 100 rpm (Supplementary Fig. S1).

### Si photodetector fabrication

Ultra-thin Si based photodetectors were fabricated using an SOI wafer with 1.25 μm thick top Si layer. The photodetectors are configured with two back-to-back photodiodes (n-p-p-n). The main fabrication steps involve selective doping to create active device and harvesting the thin device through sacrificial undercut etching of the buried oxide. Similar approach has been reported elsewhere^33^. Supplementary Fig. S9 shows the schematic fabrication steps. Specifically, 600 nm thick SiO_2_ doping mask was formed on an SOI wafer using spin on glass (700B, Filmtronics) and patterned based on photolithography and wet etching. Since the SOI wafer is slightly doped as p-type (resistivity: 11.5 Ω cm), phosphorous based spin on dopant (P510, Filmtronics) was used. The doping process was carried out at 950 °C to form the photodetectors. The top Si device was then patterned into 100 μm × 100 μm square arrays by reactive ion etching (Plasma Therm RIE, SF_6_ 40 sccm, 100 W). The wafer was immersed in buffer oxide etchant (BOE, 1: 6) for partial undercut etching of SiO_2_ for 15 min. Photoresist tethers were formed around the sides of the individual Si squares by spin coating and photolithography to prevent floating away in the following step of complete SiO_2_ removal in concentrated hydrofluoric acid (HF, concentration 49%). After completely removing the buried oxide, the photodetectors were finally picked up by a PDMS stamp.

### Si membrane preparation

The ultra-thin Si membranes (300 nm thick) with the size of 5 cm × 5 cm were harvested from a silicon-on-insulator (SOI) wafer with the top single crystal Si of 300 nm thick. The major fabrication steps involve opening releasing holes within the Si layer and undercut etching of the buried oxide to release the membrane. Specifically, the releasing holes within the top Si layer in an array form (size: 8 μm × 8 μm; period: 80 μm) were first defined by photolithography and reactive ion etching (Plasma Therm RIE, SF_6_ 40 sccm, 100 W). After the removal of the photoresist in acetone, the wafer was immersed in HF solutionfor 40 min to undercut etch the SiO_2_ through the releasing holes. The Si membrane is therefore separated from the SOI wafer and can be transfer printed onto the PCL film.

### Thin resistive heater preparation

The shadow mask was made of polyimide film (12.5 μm Kapton film, Dupont). To fabricate the shadow mask, firstly, a layer of PDMS was coated on a cleaned glass and cured to form an adhesive layer. A 12.5 µm thick polyimide film was carefully laminated onto the PDMS surface. Thereafter, a layer of 300 nm of copper, serving as etching mask for the polyimide, was deposited on top of the polyimide film using e-beam evaporation, followed by patterning through photolithography and copper wet etching processes. Reactive ion etching (oxygen: 40 sccm, power: 250 W) was performed for 8 hours to etch through the polyimide. The shadow mask was therefore completed and peeled off from the PDMS adhesive.

The metal resistor was fabricated by electron beam deposition of a layer of 1.4 μm thick silver on the PCL film through the shadow mask.

### Characterization

The optoelectronic characteristics of Si photodetectors were measured based on Keithley 4200 under a probe station. The porosity of the electrospun PCL nanofibrous polymer film was estimated based on the following Equations (3) and (4)^36^.

3 $$Density\,of\,electrospun\,PCL=\frac{Mass\,of\,electrospun\,PCL(g)}{Volume\,of\,electrospun\,PCL(c{m}^{3})};$$

4 $$Porosity( \% )=(1-\frac{Density\,of\,electrospun\,PCL(g/c{m}^{3})}{Density\,of\,PCL(g/c{m}^{3})})\times 100 \% {\rm{.}}$$

## Electronic supplementary material

Supplementary Information
