# Supplementary material for: Thermally Triggered Mechanically Destructive Electronics Based On Electrospun Poly(ε-caprolactone) Nanofibrous Polymer Films
Source: Sci Rep. 2017 Apr 19;7:947. doi: 10.1038/s41598-017-01026-6 (PMC5430441; doi:10.1038/s41598-017-01026-6)
Supplement: Supplementary file 1 — Supplementary Information [file 41598_2017_1026_MOESM1_ESM.pdf]

Supplementary Information

Thermally Triggered Mechanically Destructive Electronics Based On Electrospun Poly(ε-caprolactone) Nanofibrous Polymer Films

Yang Gao[a,b,]*, Kyoseung Sim[c], Xin Yan[a] , Jiang Jiang[d], Jingwei Xie[d], & Cunjiang Yu[a,c,e,]*

[a] Department of Mechanical Engineering, University of Houston, Houston, TX 77204 (USA)

[b] School of Mechanical and Power Engineering, East China University of Science and Technology, Shanghai 200237 (China)

[c] Materials Science and Engineering Program, University of Houston, Houston, TX 77204 (USA)

[d] Department of Surgery, Mary & Dick Holland Regenerative Medicine Program, University of Nebraska Medical Center, Omaha, NE 68198 (USA)

[e] Department of Electrical and Computer Engineering, University of Houston, Houston, TX 77204 (USA)

*Corresponding Authors, E-mail: ygao1412809@163.com; cyu15@uh.edu.

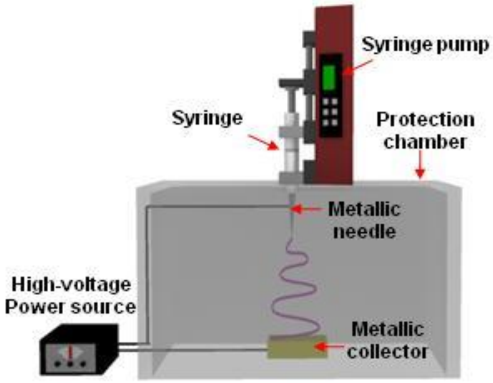

Figure S1. Schematic illustration of the experimental setup for electrospinning.

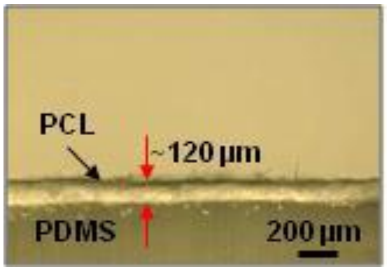

Figure S2. Optical cross-sectional image of an electrospun PCL nanofibrous polymer film.

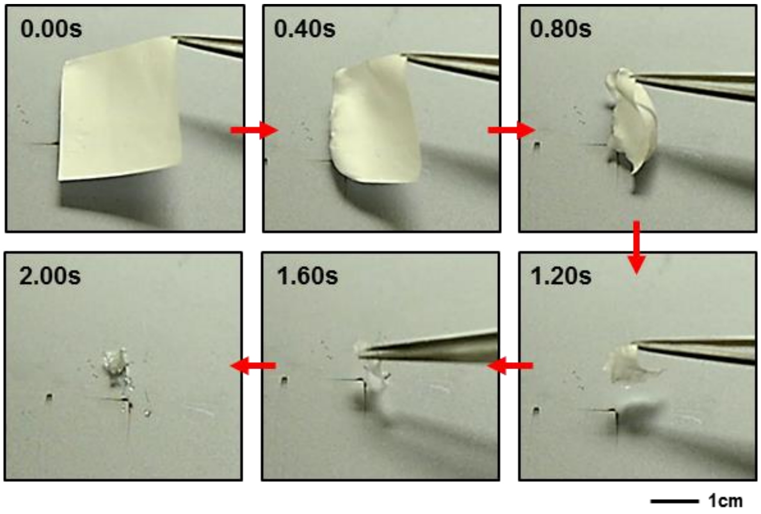

Figure S3. Sequential images of the time-lapse shrinkage process for the PCL nanofibrous film.

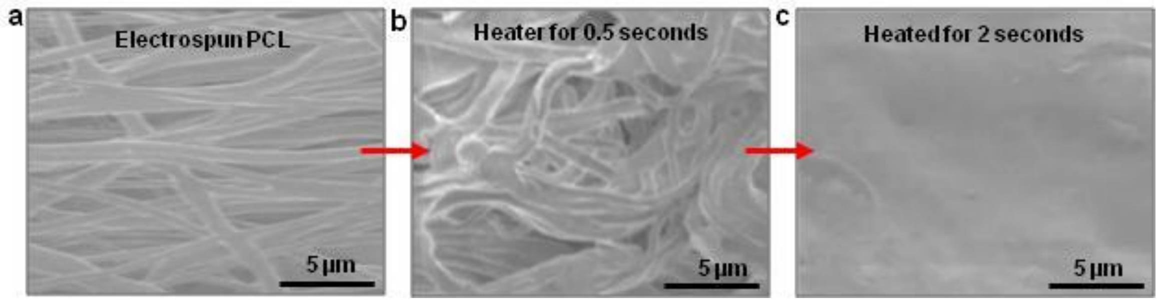

Figure S4. Time sequential SEM images of the electrospun PCL nanofibrous polymer film heated at 90 ^{o}C.

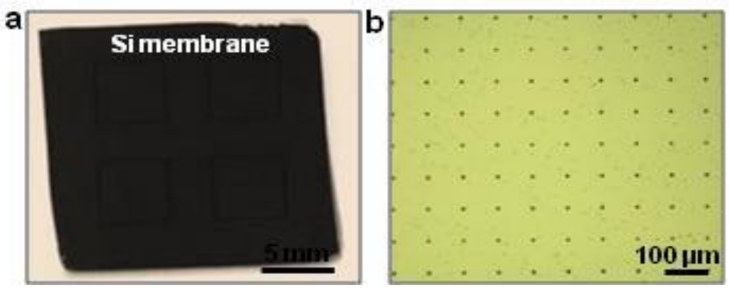

Figure S5. (a) and (b) Optical images of the Si membrane on an SOI wafer.

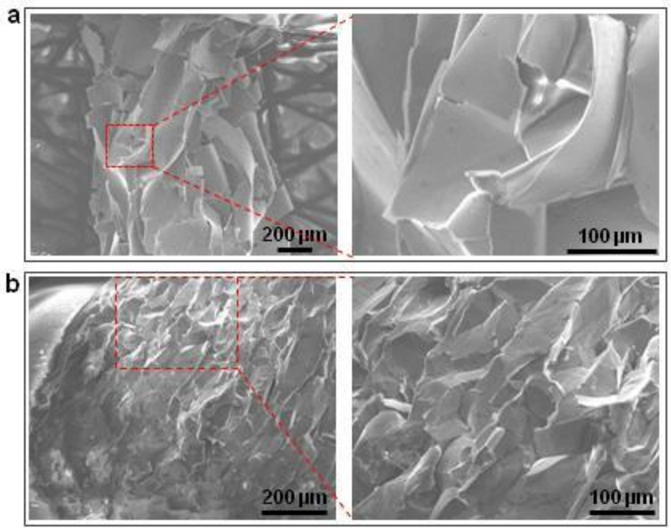

Figure S6. SEM images of the (a) 300 and (b) 100 nm thick Si membranes on an electrospun PCL nanofibrous polymer film fragmentatized by heat

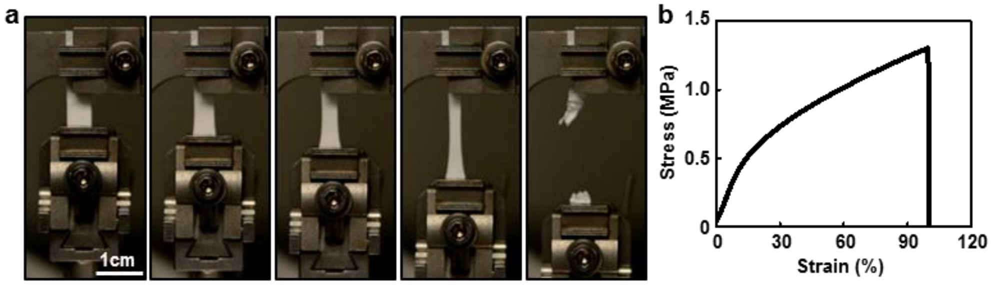

Figure S7. (a) Sequential images of PCL nanofibrous film under tensile testing. (b) The obtained Stress-Strain curve.

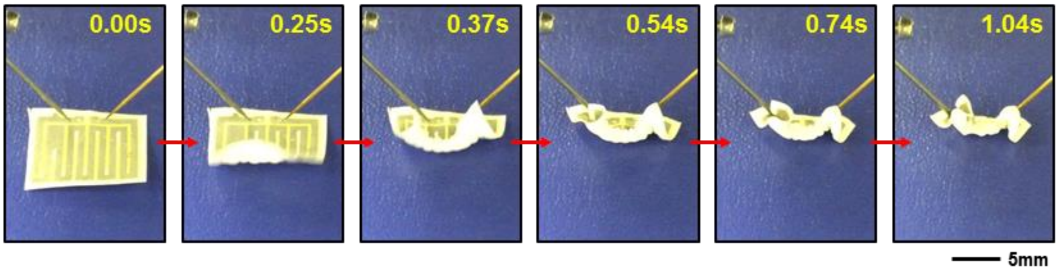

Figure S8. Sequential images of PCL nanofibrous film shrinkage triggered with a resistive heater.

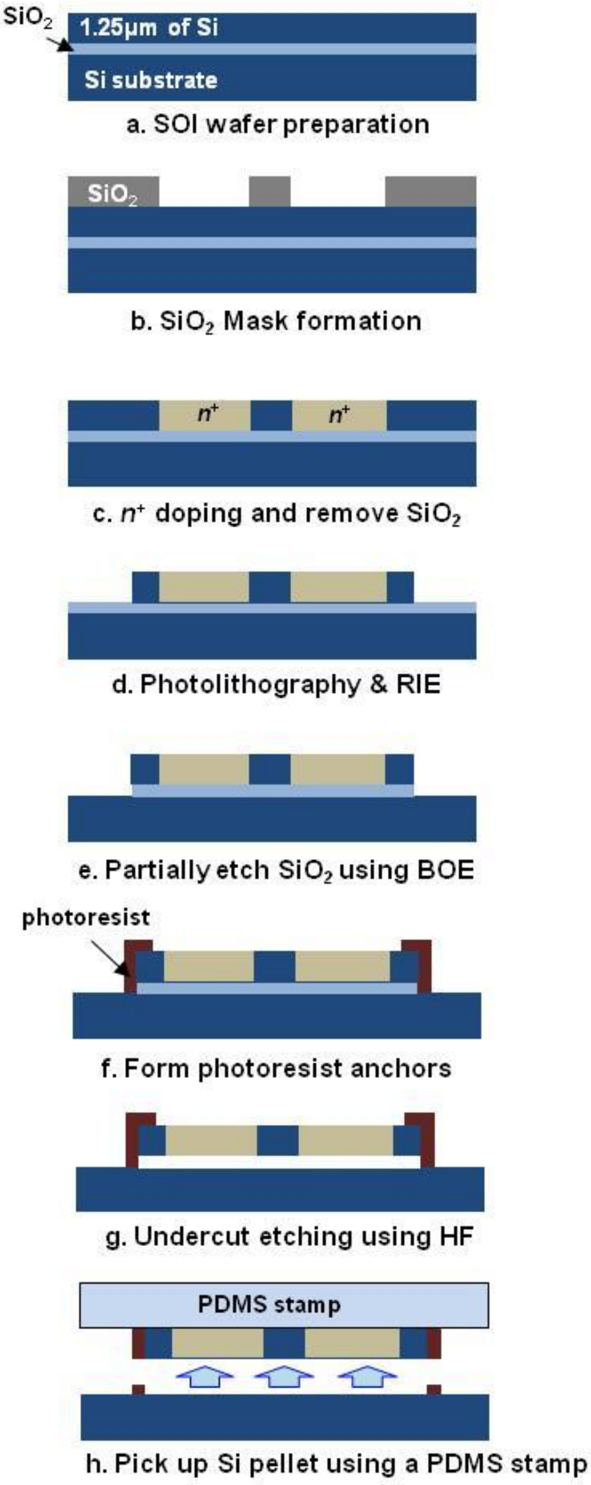

Figure S9. Schematic illustration of the device fabrication procedures for the Si based photodetectors.
